# Quantification of Islet Cell Cluster Size Heterogeneity in Early Postnatal Porcine Pancreas

**DOI:** 10.1111/xen.70164

**Published:** 2026-09-08

**Authors:** Daviana Munoz Cruz, Johanna Frick, Michel Adamina, Léo Bühler, Carmen Gonelle‐Gispert

**Affiliations:** ^1^ Surgical Research Unit, Faculty of Science and Medicine University of Fribourg Fribourg Switzerland

## Abstract

Donor scarcity limits islet transplantation for type 1 diabetes. Early postnatal piglets are a potential xenogeneic source of endocrine tissue, yet their immature islet cell clusters (ICCs) remain insufficiently characterised with respect to size and maturation. Here, we quantified ICC size heterogeneity in pancreata from six 14‐day‐old piglets using insulin and glucagon immunofluorescence staining and large‐area immunofluorescence imaging with automated whole‐section analysis. ICCs were classified as small (1–2 endocrine cells), intermediate (3–5 endocrine cells), or large (≥6 endocrine cells). Well‐developed ICCs reaching ∼100 µm in diameter were consistently identified in all animals and exhibited an intermingled β‐ and α‐cell cytoarchitecture similar to that of adult pig and human islets. The densities of small, intermediate, and large clusters were largely comparable among littermates and across piglets, suggesting a consistent distribution of ICC size classes at this postnatal age. Quantitative composition analysis revealed a strong predominance of insulin‐positive area. In small and intermediate ICCs, we observed mean β/α cell ratios of 3.7 ± 0.4 and 3.8 ± 0.4, respectively. In large ICCs, the proportion of glucagon staining was significantly higher, decreasing the β/α cell ratio to 2.2 ± 0.3. Together, these data point to a reproducible distribution of ICC sizes in 14‐day‐old piglet pancreata, including a population of morphologically well‐developed, ∼100 µm‐sized clusters. These findings from native pancreatic tissue provide a morphological basis for further evaluating 14‐day‐old piglets as a potential donor source for xenogeneic islet transplantation.

## 1 Introduction

The shortage of human donor pancreata remains a major limitation for β‐cell replacement therapies in type 1 diabetes [1]. To address this limitation, current research aims to develop unlimited sources of insulin‐producing cells, such as human embryonic stem cell‐derived islets or porcine islets [2, 3, 4]. Porcine islets represent a promising alternative source due to their physiological compatibility with humans and their practically unlimited availability [1, 5, 6]. Moreover, the development of gene‐edited pigs improves histocompatibility and reduces immunological rejection, while potentially allowing genetic modifications to enhance graft function [1, 3].

Among the different donor models investigated, early postnatal piglets have attracted particular interest due to their lower breeding costs and the relative ease with which islet cell clusters (ICCs) can be isolated and expanded compared to older pigs [1, 5, 7, 8, 9]. However, ICC development, size, and clustering dynamics at specific postnatal ages remain poorly characterized and the most suitable donor age for islet isolation remains to be defined [6, 9, 10, 11]. The existing literature reports inconsistent findings regarding endocrine maturation and ICC size, with some studies describing minimal aggregation at early ages while others report the presence of large clusters (Table 1) [2, 5, 6, 7, 8, 9, 10, 11, 12]. Moreover, because ICCs are functionally immature, most studies rely on cultured ICCs rather than native tissue, limiting direct comparisons between studies (Table 1). These discrepancies likely reflect variations in donor age, pancreatic region analysed, tissue processing, isolation procedures, culture conditions, and morphometric methodology, thereby complicating the identification of the most suitable postnatal donor age for islet xenotransplantation [6, 12, 13].

**TABLE 1:** Overview of published studies investigating ICC morphology, size heterogeneity and endocrine cell composition.

Study	Donor characteristics	Pancreatic region	Preparation	Morphology & content methods	Major morphological findings	ICC/islet size	β‐cell content (Mean ± SEM)^*^	α‐cell content (Mean ± SEM)^*^	Limitations
Korbutt, GS et al. (1996) [8]	Landrace x Yorkshire; 1–3 d; mixed sex	Whole pancreas	Fresh & cultured ICCs (0, 3, 9 d)	EM + IHC	Scattered endocrine cells (no islets) at isolation; in vitro proliferation & differentiation capacity	Majority < 150 µm after culture	5 ±1% (fresh); 24 ± 3% (cultured)	2 ±1% (fresh); 8 ±1% (cultured)	No regional/lobe‐specific analysis
Jay et al. (1999) [10]	Cotswold; 5, 12, 24 week; sex NR	SL + DL	Native tissue	IHC	Mostly scattered β‐cells at 5 week; larger islets infiltrated by exocrine tissue	Majority < 50 µm	ND	ND	No sex‐specific data; partial whole‐pancreas representation
Nielsen et al. (2003) [11]	Danish Landrace x Yorkshire; 1–3 d; sex NR	Whole pancreas	Isolated ICCs cultured up to 36 d	IHC	Random endocrine distribution initially; progressive organization during culture	Majority ≤ 150 µm	Peak ∼20% (14 d culture)	Peak ∼38% (18 d culture)	No sex‐specific data; no regional/lobe‐specific analysis
Lamb et al. (2014) [5]	Yorkshire; 4–30 d; male	Whole pancreas	Isolated ICCs cultured for 8–9 d	Light microscopy (H&E)	Full in vitro maturation achieved at day 8	∼72% at 50–100 µm (postdigestion); ∼90% at 50–150 µm (cultured)	ND	ND	No regional/lobe‐specific analysis
Krishnan et al. (2015) [9]	Yorkshire; 5–10 d, 18–22 d, 45–60 d, > 90 d; male	DL	Native tissue	Light microscopy (H&E) + IHC	Progressive endocrine aggregation with age	Mean size: 59 µm (5–10 d); 105 µm (18–22 d)	55.5 ± 3.0 % (5–10 d); 80.9 ± 2.2 % (18–22 d)	40.3 ± 4.1 % (5–10 d); 14.3 ± 1.7 % (18–22 d)	Single pancreatic lobe analysis
Hassouna et al. (2018) [2]	Duroc; 1–3 d; sex NR	Whole pancreas	Native tissue + isolated ICCs cultured for 20 d	IF	Scattered endocrine clusters/single cells before culture	NR	41.8 ± 2.0% (differentiated)	17.4 ± 0.9% (differentiated)	No sex‐specific data; no regional/lobe‐specific analysis
Nagaya et al. (2019) [6]	Large White x Landrace x Duroc; 0–180 d; mixed sex	SL + DL	Native tissue	IHC	No compact islets before 21 d; mature distribution between 28–35 d	Majority < 100 µm at 28–35 d	ND	ND	Limited tissue biopsy and fields of view
Arefanian et al. (2022) [7]	Duroc x Large White; 3, 5, 7, 10 d; male	Whole pancreas	Isolated ICCs cultured for 7 d	IHC	Similar size distribution across age groups	Majority ≤ 150 µm, with ∼70% at 50–100 µm	∼29% (3d); ∼27% (5d); ∼24% (7d); ∼35% (10d)	∼28% (3 d); ∼19% (5 d); ∼19% (7 d); ∼23% (10 d)	No regional/lobe‐specific analysis
Seeberger et al. (2023) [12]	Duroc x Landrace; 1–3 d; mixed sex	SL + DL + CL	Native tissue + isolated ICCs cultured for 15 d	IF	Heterogeneity among lobes (notably endocrine content & ICC size)	Mean size: 3 d culture: DL (98.7 µm) < CL (135.8 µm) ≈ SL (137.9 µm); 15 d culture: CL (117.8 µm) < DL (136.7 µm) ≈ SL (144.4 µm)	β/α ratio in ICCs: DL (1.5:1), CL (2:1), SL (1:1)		/
Present study	Large White x Landrace, 14 d, male	Unspecified regional origin of pancreatic sections	Native tissue	Large area IF	Well‐developed ICCs across all animals; consistent size distribution among littermates	Largest ICCs ∼100 µm	Mean β/α ratio: 3.1 ± 0.2 (Mean ± SEM); increased α‐cell proportion in larger ICCs		No stereological quantification; unknown regional origin of tissue sections

This table summarises study characteristics, methodology, major morphological findings, reported ICC/islet size, β‐ and α‐cell content, and study limitations.

Abbreviations: CL: Connecting lobe; DL: Duodenal lobe; EM: Electron microscopy; ICC: Islet cell cluster; IF: Immunofluorescence; IHC: Immunnohistochemistry; ND: Not determined; NR: Not reported; SL: Splenic lobe.

^*^ Data from Nielsen et al. presented as Median ± CI.

In a previous study, we qualitatively observed heterogeneity in ICC size distribution within early postnatal porcine pancreata; however, no dedicated quantitative analysis was performed [14]. Given the scarcity of systematic data on ICC development in native pancreatic tissue, the present study aimed to investigate ICC size heterogeneity and endocrine composition in the pancreas of 14‐day‐old piglets. Specifically, we used automated fluorescent image analysis in NIS‐Elements (GA3) to quantitatively assess ICC size distribution and β/α cell ratios across large tissue sections, enabling a broader tissue‐level assessment that may reduce sampling bias compared with analyses restricted to selected microscopic fields.

## 2 Materials and Methods

### 2.1 Immunofluorescence Staining on Porcine Pancreas Sections

Six 14 (±1)‐day‐old Large White × Landrace pig pancreata (Appel, Switzerland) from two litters were analysed. Formalin‐fixed, paraffin‐embedded tissues were sectioned (4 µm, non‐consecutive), yielding 24 pancreatic sections from six male pigs. Immunofluorescence staining was performed as previously described [14] using antibodies against insulin (DAKO, #A0564), glucagon (Abcam, #ab92517), and either vimentin (DAKO, #M7020) or PCNA (Biotium, #0467). Secondary antibodies comprised coumarin AMCA‐conjugated (Jackson ImmunoResearch, #706–155–148), Alexa555‐conjugated (Invitrogen, #A21428), and Alexa488‐conjugated (Jackson ImmunoResearch, #715–546–151) antibodies.

Large fluorescent images capturing the entire pancreatic tissue sample were acquired using a Nikon Eclipse Ni microscope. The scan area and a focus map were defined for each section to ensure consistent image quality. Acquisition settings were kept constant across samples, and Look‐Up Tables (LUTs) were adjusted to minimise background and enhance signal.

### 2.2 Workflow Design and Analysis of the Porcine Pancreatic Sections

Automated counting of ICC by size category and quantification of endocrine composition were performed in NIS‐Elements (Nikon). The first workflow was established to delineate pancreatic tissue and detect insulin‐ and glucagon‐positive cells using thresholding and morphological processing (Figure S1). Adjacent endocrine‐positive regions were merged to define individual ICCs, which were classified as small (1–2 cells), intermediate (3–5 cells), or large (≥6 cells) (Figure S2 and S3). Insulin and glucagon staining percentages were also quantified in small clusters. To account for morphological variability and section defects, segmentation parameters were adjusted slightly before analysis in a blinded manner, and false positives (e.g., fluorescent artefacts) were manually excluded.

A second workflow was used to quantify insulin‐ and glucagon‐positive areas within intermediate and large clusters and across entire sections based on the binary layers defined during the first analysis (Figure S4). Regions of interest for individual clusters were established based on fluorescence intensity; the insulin‐positive area was measured and normalized to total cluster area, with the remaining area attributed to glucagon. Although this method does not explicitly account for minor unstained cell populations, unlabeled regions within ICCs were rarely detected; therefore, the associated bias was considered negligible.

### 2.3 Statistical Analysis

Statistical analyses were performed using GraphPad Prism (two‐way ANOVA). All data are presented as Mean ± SEM. A *p*‐value of *p* < 0.05 was considered statistically significant.

One section in pancreas group 1 was excluded from the β/α ratio analysis because the near‐zero glucagon‐positive area in that section (Figure 3C) resulted in an unreliable ratio (β/α ratio = 84).

## 3 RESULTS

### 3.1 Early Postnatal Porcine Pancreata Contain 100 µm‐large, Developed ICCs

**FIGURE 1:**
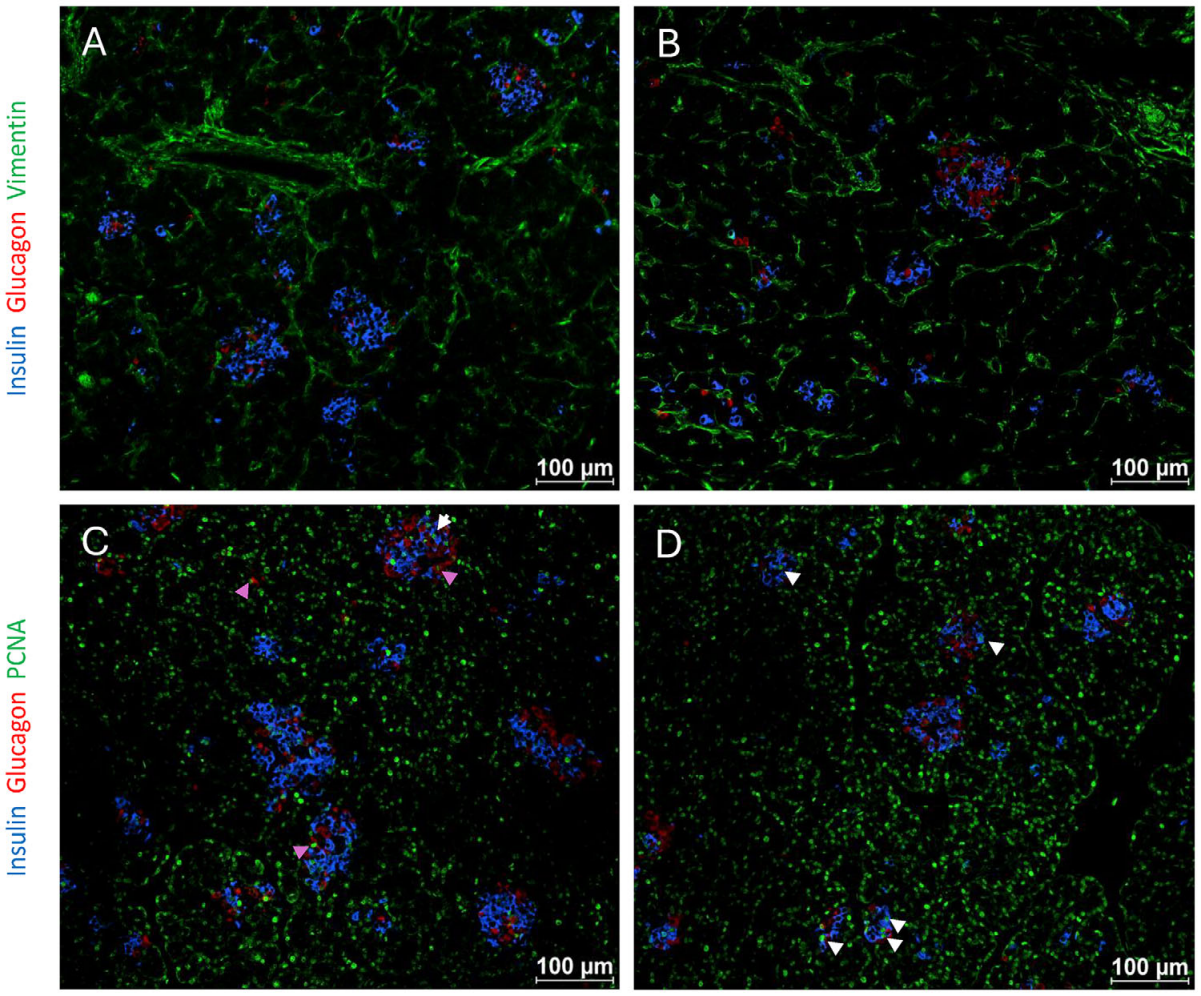
Immunostaining of large ICCs in early postnatal pig pancreas. Immunofluorescence staining of ICCs showing insulin‐positive β‐cells (blue) and glucagon‐positive α‐cells (red). A, B) Vimentin‐positive mesenchymal cells (green) highlight the surrounding stromal tissue. C, D) PCNA immunostaining (green) shows proliferating ductal and endocrine cells, including α‐cells (magenta arrows) and β‐cells (white arrows). Smaller ICCs and individual stained cells are also visible. Scale bars: 100 µm.

An earlier study of 14‐day‐old piglet pancreas sections suggested heterogeneous ICC development within and between litters [14]. Immunostaining of α‐ and β‐cells revealed ICCs of varying sizes across all samples. While small and intermediate clusters predominated, large, well‐developed ICCs were present in all sections (Figure 1). These larger ICCs displayed intermingled β‐ and α‐cells, resembling adult porcine and human islets [15, 16]. The largest were circular to oval, reaching diameters of ∼100 µm. Vimentin co‐staining revealed supportive stromal tissue, with endocrine cells adjacent to vimentin‐positive cells (Figures 1A, 1B). PCNA staining showed high proliferation in exocrine and ductal cells but limited endocrine cell proliferation; the latter was mostly observed in larger ICCs, suggesting ongoing low‐level postnatal β‐ and α‐cell proliferation at this early postnatal stage (Figures 1C, 1D).

### 3.2 The Number of ICCs of Different Sizes Appears Consistent among the Six Early Postnatal Piglets

**FIGURE 2:**
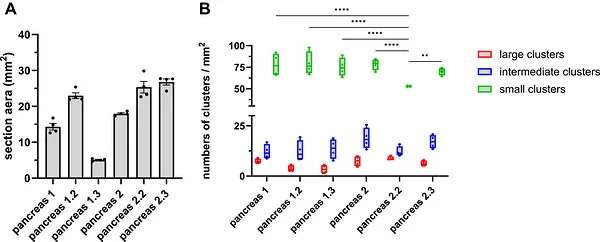
Quantification of small, intermediate, and large ICCs in whole‐section images of pancreatic tissue. A) Mean area of all tissue sections analysed, expressed in mm^2^. B) Mean density of small, intermediate and large ICCs per mm^2^ across the six pancreatic tissue sections. Four whole tissue sections were analysed per pancreas (*n* = 6 pancreas). Data are presented as Mean ± SEM. * *p* ≤ 0.05; ** *p* ≤ 0.01; *** *p* ≤ 0.001; **** P ≤ 0.0001.

ICC heterogeneity was quantified using automated image analysis (NIS‐Elements) on non‐consecutive sections. As the analysed pancreatic sections differed in size (Figure 2A), ICC size distribution initially appeared to vary between samples (data not shown). However, after tissue area normalisation, these interindividual differences were not statistically significant, indicating consistent ICC size distributions across the 14‐day‐old piglets (Figure 2B). Mean densities (ICCs/mm^2^; mean ± SEM) were 72.2 ± 4.1 (small), 14.4 ± 2.1 (intermediate), and 6.2 ± 0.8 (large) (Table 2). Large and intermediate ICC densities were comparable across samples, with only one exception showing a significant decrease in the proportion of small ICCs (Figure 2B).

**TABLE 2:** Density of islet cell clusters of different sizes in early postnatal pig pancreatic sections.

	Large clusters		Intermediate clusters		Small clusters	
	Density per mm^2^	Proportion (%)	Density per mm^2^	Proportion (%)	Density per mm^2^	Proportion (%)
**Pancreas 1**	7.4 ± 0.6	7.8 ± 0.3	12.1 ± 1.8	12.3 ± 0.6	77.9 ± 6.6	80.3 ± 0.8
**Pancreas 1.2**	3.7 ± 0.8	3.8 ± 0.5	12.3 ± 2.6	12.8 ± 1.4	79.3 ± 6.8	83.8 ± 1.7
**Pancreas 1.3**	3.4 ± 1.1	3.8 ± 0.9	13.6 ± 2.5	14.3 ± 1.7	75.2 ± 5.4	82.0 ± 2.3
**Pancreas 2**	7.4 ± 1.3	7.0 ± 0.9	18.8 ± 2.6	17.8 ± 1.5	78.2 ± 3.3	75.3 ± 2.1
**Pancreas 2.2**	8.8 ± 0.5	12.0 ± 0.4	12.2 ± 1.3	16.5 ± 1.3	52.9 ± 0.5	71.8 ± 1.7
**Pancreas 2.3**	6.3 ± 0.7	6.8 ± 0.5	17.1 ± 1.7	18.3 ± 1.0	69.7 ± 2.1	75.0 ± 1.5
**Mean**	**6.2 ± 0.8**	**6.9 ± 0.6**	**14.4 ± 2.1**	**15.3 ± 1.3**	**72.2 ± 4.1**	**78.0 ± 1.6**

ICC counts across size categories were normalised to tissue section area and expressed per square millimetre of tissue (number/mm^2^).

Relative percentage proportions for each size category are also indicated. Four whole tissue sections were analysed per pancreas (*n* = 6 pancreata). Data are presented as Mean ± SEM.

### 3.3 Insulin Expression Is Predominant across Different‐sized ICCs and in the Whole Pancreatic Sections

**FIGURE 3:**
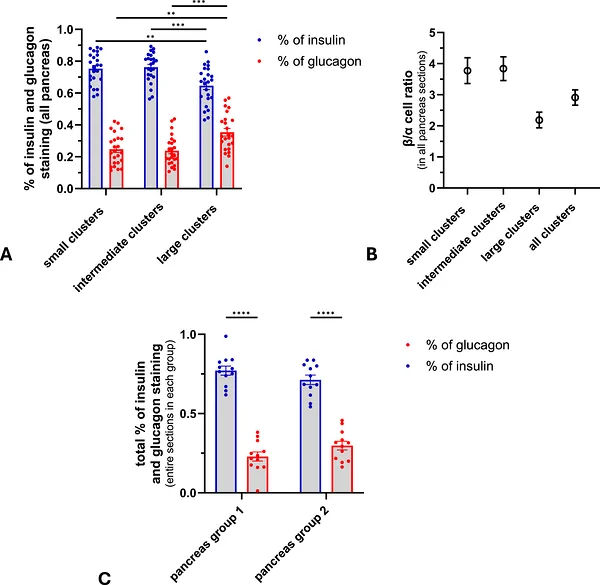
Insulin and glucagon proportion in ICCs of different sizes and in entire pancreatic sections. A) Percentage of insulin and glucagon staining across small, intermediate, and large ICCs, pooling pancreatic sections from both littermate groups. B) Mean β/α cell ratios across different‐sized ICCs, as well as in all ICCs combined. C) Total percentage of insulin‐ and glucagon‐positive area across whole pancreatic tissue sections for each group of littermates. Data are presented as Mean ± SEM. * *p* ≤ 0.05; ** *p* ≤ 0.01; *** *p* ≤ 0.001; **** P ≤ 0.0001.

Insulin staining predominated over glucagon across all ICC sizes in all sections of both littermate groups. Data from all sections were pooled, and the mean total percentages of insulin and glucagon staining were determined (Figure 3A). The corresponding β/α cell ratios across different‐sized ICCs were calculated. Across all 14‐day‐old porcine pancreatic sections, the overall β/α cell ratio across all ICCs was 3.1 ± 0.2 (mean ± SEM). However, large ICCs contained significantly more glucagon‐positive α‐cells, resulting in a lower β/α ratio (2.2 ± 0.3) than small (3.7 ± 0.4) and intermediate (3.8 ± 0.4) ICCs (Figure 3B).

Comparison of the percentage of insulin‐ and glucagon‐stained cells between the two littermate groups revealed no significant differences. In Group 1, insulin‐positive cells accounted for 77.1 ± 0.03 % (mean ± SEM) of endocrine cells, yielding a β/α cell ratio of 3.4 ± 0.4. Similarly, Group 2 contained 71.3 ± 0.03 % insulin‐positive cells (Figure 3C), resulting in a β/α ratio of 2.7 ± 0.3.

## 4 Discussion

Early postnatal piglets are a promising source of ICCs, but the optimal porcine donor model remains undefined. Here, we quantitatively analysed ICC size distribution and endocrine composition in 14‐day‐old piglet pancreata using automated, large‐area fluorescent image analysis. The functional properties and molecular maturation of ICCs of different sizes were beyond the scope of this study and will require further investigation. The quantitative ICC analysis revealed consistent ICC size distributions across all samples, suggesting that α‐ and β‐cell organisation is relatively reproducible at postnatal day 14. As summarized in Table 1, published studies differ substantially in donor age, pancreatic region analysed, tissue preparation (native tissue versus isolated and cultured ICCs), and imaging methodology. These methodological differences likely contribute to the variability reported in early postnatal ICC morphology and endocrine composition. In the present study, relatively large ICCs (∼100 µm in diameter) were consistently observed in all animals analysed, which contrasts with some previous studies of native pancreatic tissue [6, 10]. The use of large‐area whole‐section analysis may have reduced bias introduced by microscopic field selection and may partly explain discrepancies with earlier reports (Table 1).

Insulin‐positive cells predominated across all ICC size categories. However, larger ICCs contained relatively more glucagon‐positive cells than small and intermediate ICCs, resulting in a lower β/α ratio. Although the functional significance of this observation remains undetermined, the predominance of insulin‐positive cells is consistent with the β‐cell‐rich composition described in the adult porcine pancreas [17].

Beyond its translational relevance, the porcine pancreas provides a valuable model for investigating postnatal endocrine development. To complement the quantitative ICC analysis, we qualitatively evaluated PCNA and vimentin immunostaining at postnatal day 14. Although these observations were not quantified, PCNA staining revealed proliferating endocrine cells, predominantly within larger ICCs, whereas vimentin staining demonstrated a close association between endocrine cells and surrounding mesenchymal structures. These observations in piglets are consistent with previous descriptions of mesenchymal‐endocrine interactions in rodents [18, 19, 20].

The present study provides a quantitative two‐dimensional assessment of ICC morphology. However, it does not capture total endocrine cell mass or the three‐dimensional spatial distribution of ICCs throughout the pancreas. Regional differences in ICC size and endocrine composition across the pancreas have been reported [12]. Although tissue samples were collected randomly from pancreata including all three lobes, the lobe of origin of the individual sections was not recorded. Consequently, regional differences could not be assessed. Nevertheless, the consistent ICC distributions observed across randomly sampled tissues from all animals suggest that the principal findings are unlikely to be explained solely by regional sampling bias. Future studies employing systematic lobe‐specific sampling will be required to fully address regional endocrine heterogeneity. Although sex‐related differences have been reported following neonatal porcine islet transplantation [21], previous morphological studies, including those using mixed‐sex animals (Table 1), either did not analyse native ICC morphology or revealed only limited sex‐related differences [12]. However, future studies including female piglets will be important to confirm whether our findings apply to both sexes.

Future deep‐tissue three‐dimensional imaging approaches could address these limitations by providing a complete mapping of endocrine cell distribution while preserving the spatial organisation of ICCs. Such approaches have revealed unexpected heterogeneity in human islet size and composition [22], and could provide a valuable reference for evaluating the efficiency of early postnatal ICC isolation procedures.

Current ICC isolation protocols rely on collagenase digestion and mechanical dissociation. Variability in enzyme delivery and tissue digestion may differentially affect ICC recovery, fragment larger clusters, and thereby alter the apparent size distribution of isolated ICCs [23]. Consequently, the ICC morphology observed in situ is not directly comparable to that of isolated or cultured ICC preparations, and it remains to be determined whether modified isolation protocols can allow recovery of larger ICCs.

In conclusion, 14‐day‐old porcine pancreata appear to contain consistent populations of ICCs of different sizes, including large, morphologically developed clusters approaching 100 µm in diameter. These findings provide a quantitative morphological reference for future studies investigating early postnatal porcine pancreatic development and the optimisation of ICC isolation.

## Supporting information




**Figure S1:** GA3 workflow 1 for pancreatic section delineation and ICC identification. **Figure S2:** Example of a segmented and delineated pancreatic section after using workflow 1. **Figure S3:** Example of software recognition of ICCs of different sizes. **Figure S4:** GA3 workflow 2 for quantification of hormone‐positive areas.

## References

[1] D. K. C. Cooper , L. Mou , and R. Bottino , “A Brief Review of the Current Status of Pig Islet Xenotransplantation,” Frontiers in immunology 15 (2024): 1366530, 10.3389/fimmu.2024.1366530. 38464515 PMC10920266

[2] T. Hassouna , K. L. Seeberger , B. Salama , and G. S. Korbutt , “Functional Maturation and in Vitro Differentiation of Neonatal Porcine Islet Grafts,” Transplantation 102, no. 10 (2018): e413–e423, 10.1097/TP.0000000000002354. 29975241

[3] Z. Liu , W. Hu , T. He , et al., “Pig‐to‐Primate Islet Xenotransplantation: Past, Present, and Future,” Cell Transplantation 26, no. 6 (2017): 925–947, 10.3727/096368917X694859. 28155815 PMC5657750

[4] T. W. Reichman , J. F. Markmann , J. Odorico , et al., “Stem Cell–Derived, Fully Differentiated Islets for Type 1 Diabetes,” New England Journal of Medicine 393, no. 9 (2025): 858–868, 10.1056/NEJMoa2506549. 40544428

[5] M. Lamb , K. Laugenour , O. Liang , M. Alexander , C. E. Foster , and J. R. Lakey , “In Vitro Maturation of Viable Islets from Partially Digested Young Pig Pancreas,” Cell Transplantation 23, no. 3 (2014): 263–272, 10.3727/096368912X662372. 23394130

[6] M. Nagaya , A. Hayashi , K. Nakano , et al., “Distributions of Endocrine Cell Clusters during Porcine Pancreatic Development,” PLoS ONE 14, no. 5 (2019): e0216254, 10.1371/journal.pone.0216254. 31075154 PMC6510474

[7] H. Arefanian , Q. Ramji , N. Gupta , et al., “Yield, Cell Composition, and Function of Islets Isolated from Different Ages of Neonatal Pigs,” Frontiers in Endocrinology 13 (2022): 1032906, 10.3389/fendo.2022.1032906. 36619563 PMC9811407

[8] G. S. Korbutt , J. F. Elliott , Z. Ao , D. K. Smith , G. L. Warnock , and R. V. Rajotte , “Large Scale Isolation, Growth, and Function of Porcine Neonatal Islet Cells,” Journal of Clinical Investigation 97, no. 9 (1996): 2119–2129, 10.1172/JCI118649. 8621802 PMC507287

[9] R. Krishnan , N. Truong , M. Gerges , et al., “Impact of Donor Age and Weaning Status on Pancreatic Exocrine and Endocrine Tissue Maturation in Pigs,” Xenotransplantation 22, no. 5 (2015): 356–367, 10.1111/xen.12184. 26381493

[10] T. R. Jay , K. A. Heald , N. J. Carless , D. E. Topham , and R. Downing , “The Distribution of Porcine Pancreatic Beta‐cells at Ages 5, 12 and 24 Weeks,” Xenotransplantation 6, no. 2 (1999): 131–140. 10431790 10.1034/j.1399-3089.1999.00009.x

[11] T. B. Nielsen , K. B. Yderstraede , H. D. Schrøder , J. J. Holst , K. Brusgaard , and H. Beck‐Nielsen , “Functional and Immunohistochemical Evaluation of Porcine Neonatal Islet‐like Cell Clusters,” Cell Transplantation 12, no. 1 (2003): 13–25, 10.3727/000000003783985142. 12693660

[12] K. L. Seeberger , B. F. Salama , S. Kelly , et al., “Heterogenous Expression of Endocrine and Progenitor Cells within the Neonatal Porcine Pancreatic Lobes–Implications for Neonatal Porcine Islet Xenotransplantation,” Xenotransplantation 30, no. 2 (2023): e12793, 10.1111/xen.12793. 36748727

[13] S. Nagaraju , R. Bottino , M. Wijkstrom , M. Trucco , and D. K. C. Cooper , “Islet Xenotransplantation: What Is the Optimal Age of the Islet‐Source Pig?,” Xenotransplantation 22, no. 1 (2015): 7–19, 10.1111/xen.12130. 25130196

[14] E. Montanari , L. Szabó , A. Balaphas , et al., “Multipotent Mesenchymal Stromal Cells Derived from Porcine Exocrine Pancreas Improve Insulin Secretion from Juvenile Porcine Islet Cell Clusters,” Xenotransplantation 28, no. 3 (2021): e12666, 10.1111/xen.12666. 33538027

[15] O. Cabrera , D. M. Berman , N. S. Kenyon , C. Ricordi , P. O. Berggren , and A. Caicedo , “The Unique Cytoarchitecture of human Pancreatic Islets Has Implications for Islet Cell Function,” Proceedings of the National Academy of Sciences 103, no. 7 (2006): 2334–2339, 10.1073/pnas.0510790103. PMC1413730 16461897

[16] D.‐T. Hoang , H. Matsunari , M. Nagaya , et al., “A Conserved Rule for Pancreatic Islet Organization,” PLoS ONE 9, no. 10 (2014): e110384, 10.1371/journal.pone.0110384. 25350558 PMC4211668

[17] S. Tritschler , M. Thomas , A. Böttcher , et al., “A Transcriptional Cross Species Map of Pancreatic Islet Cells,” Molecular Metabolism 66 (2022): 101595, 10.1016/j.molmet.2022.101595. 36113773 PMC9526148

[18] M. Attali , V. Stetsyuk , A. Basmaciogullari , et al., “Control of β‐Cell Differentiation by the Pancreatic Mesenchyme,” Diabetes 56, no. 5 (2007): 1248–1258, 10.2337/db06-1307. 17322477

[19] B. Duvillié , M. Attali , A. Bounacer , P. Ravassard , A. Basmaciogullari , and R. Scharfmann , “The Mesenchyme Controls the Timing of Pancreatic β‐Cell Differentiation,” Diabetes 55, no. 3 (2006): 582–589, 10.2337/diabetes.55.03.06.db05-0839. 16505219

[20] D. Hibsher , A. Epshtein , N. Oren , and L. Landsman , “Pancreatic Mesenchyme Regulates Islet Cellular Composition in a Patched/Hedgehog‐Dependent Manner,” Scientific Reports 6 (2016): 38008, 10.1038/srep38008. 27892540 PMC5125096

[21] N. Cuesta‐Gomez , C. Castro , M. Rosko , K. Seeberger , and G. S. Korbutt , “Sex Differences in Maturation and Function of Neonatal Porcine Islets Upon Transplantation in Mice,” Xenotransplantation 32, no. 2 (2025): e70039, 10.1111/xen.70039. 40243327 PMC12005065

[22] J. Lehrstrand , W. I. L. Davies , M. Hahn , O. Korsgren , T. Alanentalo , and U. Ahlgren , “Illuminating the Complete Ss‐cell Mass of the human Pancreas‐ signifying a New View on the Islets of Langerhans,” Nature Communications 15, no. 1 (2024): 3318, 10.1038/s41467-024-47686-7. PMC11024155 38632302

[23] R. W. Holdcraft , M. L. Green , A. G. Breite , et al., “Optimizing Porcine Islet Isolation to Markedly Reduce Enzyme Consumption without Sacrificing Islet Yield or Function,” Transplantation Direct 2, no. 7 (2016): e86, 10.1097/TXD.0000000000000599. 27830180 PMC5087567

